# Taxas de Mortalidade por Doenças Cardiovasculares e Câncer na População Brasileira com Idade entre 35 e 74 Anos, 1996-2017

**DOI:** 10.36660/abc.20200233

**Published:** 2021-08-09

**Authors:** Antonio de Padua Mansur, Desiderio Favarato

**Affiliations:** 1 Universidade de São Paulo Faculdade de Medicina Hospital das Clínicas São PauloSP Brasil Instituto do Coração do Hospital das Clínicas da Faculdade de Medicina da Universidade de São Paulo, São Paulo, SP – Brasil

**Keywords:** Doenças Cardiovasculares/mortalidade, Epidemiologia, Mortalidade, Brasil, Doenças cerebrovasculares, Neoplasias, Isquemia Miocárdica

## Abstract

**Fundamento:**

As doenças cardiovasculares (DCV) e câncer são as principais causas de morte no mundo. Essas doenças apresentam muitos fatores de risco em comum, e o controle de fatores de risco tradicionais para DCV foi associado com menor incidência de câncer.

**Objetivo:**

Analisar tendências nas taxas de mortalidade por câncer na população brasileira com idade entre 35 e 74 anos de 1996 a 2017.

**Métodos:**

As tendências nas taxas de mortalidade (bruta e ajustada por idade) foram analisadas quanto a todas as causas de morte, DCV e câncer. Os dados foram obtidos do banco de dados de mortalidade do Ministério da Saúde. O programa Joinpoint Regression foi usado para análise das tendências e ajustes nas taxas de saúde. O grau de mudanças nas taxas foi determinado pela variação percentual anual média (VPAM). O nível de significância estatística foi estabelecido em p<0,05.

**Resultados:**

A mortalidade por todas as causas (VPAM=-1,6%; p<0,001), CVD (VPAM =-2,3; p<0,001), doenças isquêmicas do coração (DIC) (VPAM =-1,6; p<0,001) e doenças cerebrovasculares (DCbV) (VPAM =-3.7; p<0.001) diminuiu. As mesmas tendências foram observadas para DCV em homens e mulheres (p<0,001). As taxas de mortalidade por todos os tipos de câncer (AAPC=-0,1; p=0,201), em homens (VPAM =-0,1; p=0,193) e mulheres (VPAM =-0,1; p=0,871) permaneceram inalteradas. Em 2002, a mortalidade por câncer excedeu a soma de mortes por DIC e DCbV. Se as tendências continuarem, a mortalidade por câncer também excederá a mortalidade por DCV até 2024. Nas mulheres, a mortalidade por câncer de mama, pulmão e cólon, aumentou, e a mortalidade por câncer de colo de útero e de estômago diminuiu. Nos homens, a mortalidade por câncer de pulmão, estômago, e esôfago diminuiu, e por câncer de próstata permaneceu inalterada.

**Conclusão:**

As DCV são atualmente as principais causas de morte no Brasil, mas as taxas de morte por câncer irão superar as taxas por DCV em poucos anos.

## Introdução

As doenças cardiovasculares (DCV) e as neoplasias são as principais causas de morte no Brasil e no mundo.^1^^,^^2^ Em 2017, as doenças crônicas não transmissíveis (DCNT) foram responsáveis por 73,4% das mortes no mundo.^2^ Acredita-se que mais de 85% das mortes prematuras por DCNT de pessoas com idade entre 30 e 69 anos ocorreram em países de baixa renda.^3^ Doenças isquêmicas do coração (DIC) e doenças cerebrovasculares (DCbV) foram responsáveis por 60% das mortes por DCV. Um estudo prévio conduzido no Brasil apresentou uma tendência de diminuição da mortalidade por DCV de 1980 a 2012.^4^ Durante esse período, a mortalidade por DCbV reduziu de maneira mais significativa que a mortalidade por DIC. A mortalidade por DCV teve importante variações regionais no Brasil, com taxas mais altas nas regiões sul e sudeste,^5^ e convergência das taxas de mortalidade por DIC e DCbV nas cinco regiões. A convergência das taxas de mortalidade nessas regiões ocorreu mais cedo para DCbV, ao redor do ano de 1999, e mais tarde para DIC, no ano de 2007.

De acordo com a Organização Mundial da Saúde (OMS), câncer foi a segunda principal causa de morte por DCNT na população mundial.^3^ Em muitos países desenvolvidos, câncer foi a principal causa de morte em adultos com idade inferior a 70 anos. Nos EUA, a taxa de mortalidade por câncer foi mais alta que a mortalidade por DCV no grupo etário de 45 a 64 anos de 1999 a 2017,^6^ e diminuiu em 27% de 1991 a 2016. De 2007 a 2016, a redução anual foi de 1,4% nas mulheres e de 1,8% nos homens.^7^ No Brasil, câncer foi a segunda principal causa de morte em 2017.^1^

As DCV e câncer apresentam alguns fatores de risco em comum. Os principais fatores de risco para DCV estão também associados com uma maior incidência de câncer. Uma metanálise recente mostrou que cada fator de risco para DCV – tabagismo, hipertensão, diabetes, obesidade, consumo excessivo de álcool, sedentarismo e baixo nível socioeconômico, foi associado com maior incidência de câncer.^8^ Por outro lado, o controle dos principais fatores de risco para DCV associou-se com uma redução significativa na incidência de câncer.^9^ Assim, o controle dos fatores de risco para DCV tem um impacto significativo na redução de taxa de mortalidade por câncer.

Este estudo analisou tendências nas taxas de morte por todas as causas, por DCV, DIC, DCbV e câncer em mulheres e homens da população brasileira entre 1996 e 2017.

## Métodos

Nós analisamos tendências de taxa de morte por todas as causas, DCV, DIC, DCbV e câncer em homens e mulheres brasileiros entre 1996 e 2017. A taxa de mortalidade por 100 000 pessoas foi avaliada a cada cinco anos no grupo etário entre 35 e 74. A taxa de mortalidade ajustada por idade (por 100 000 pessoas) foi calculada para esse grupo etário para o período do estudo (1996-2017) usando-se o método direto estabelecido pela OMS (2000). Os dados de mortalidade foram obtidos da Estatística Vital do DATASUS do Ministério da Saúde, disponível online em www2.datasus.gov.br.^10^ As causas de morte foram classificadas segundo Classificação Internacional de Doenças (CID), 10ª revisão. As DCV foram agrupadas em códigos I00 a I99, a DIC em códigos entre I20 e I25, DCbV em códigos entre I60 e I69, e câncer em códigos entre C00 e C97. Os códigos usados para câncer de pulmão, estômago, próstata, esôfago, cólon, mama, e cervical foram, respectivamente: C34, C15, C61, C15, C18, C50 e C53. As cinco causas principais de morte foram analisadas em mulheres e homens no período entre 1996 e 2017.

### Análise estatística

Utilizamos o programa *Joinpoint Regression Program* versão 4.7.0.0 da Divisão de Controle de Câncer e Estudos Populacionais do Instituto Nacional do Câncer (*National Cancer Institute, Division of Cancer Control and Population Sciences*) para análise de tendências da taxa de morte ajustada por idade.^11^ A análise por *joinpoint* foi usada para identificar os anos (variável independente) no qual mudanças significativas na taxa de mortalidade (variável dependente) ocorreram durante o período de estudo. A intensidade das mudanças foi determinada pela variação percentual anual média (VPAM). As inclinações das linhas de regressão de DCV versus câncer e DIC versus DCbV foram comparadas pelo programa Microsoft Excel 2010 usando a estatística t e a distribuição t bicaudal.^12^ O nível de significância estatística foi estabelecido em p<0,05. O estudo não necessitou de aprovação por comitê de ética uma vez que os dados de mortalidade foram obtidos de um *website* público, e não se conhecia a identidade dos participantes.

## Resultados

**Figura 1:**
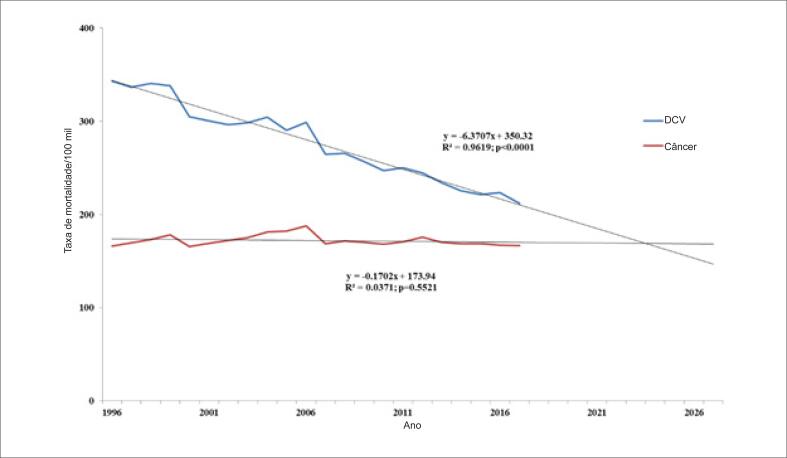
Tendências nas taxas de mortalidade por doenças cardiovasculares (DCV) e câncer na população brasileira com idade entre 35 e 74 anos, entre 1996 e 2017.

A taxa de mortalidade ajustada por idade para DCV e câncer por 100 000 pessoas correspondeu a 50% das mortes por todas as causas. DCV e câncer foram responsáveis por aproximadamente 30% e 20% da mortalidade total, respectivamente. A mortalidade por DCV diminuiu em 38% de 1996 a 2017, e a mortalidade por câncer permaneceu a mesma (p<0,01 para as comparações das inclinações das linhas de regressão das taxas de mortalidade ajustada pela idade por DCV e câncer). Em 1996, a mortalidade por câncer foi 52% menor que a mortalidade por DCV, mas 22% mais baixa em 2017. Se essas tendências continuarem, a mortalidade por câncer será igual à mortalidade por DCV no início de 2024 (Figura 1). Igualmente, a taxa bruta de mortalidade analisada a cada cinco anos no período entre 35 e 74 anos mostrou que a mortalidade por DCV foi sempre mais alta que a taxa de mortalidade por câncer (Tabela 2).

### Mortalidade por todas as causas

**Figura 2:**
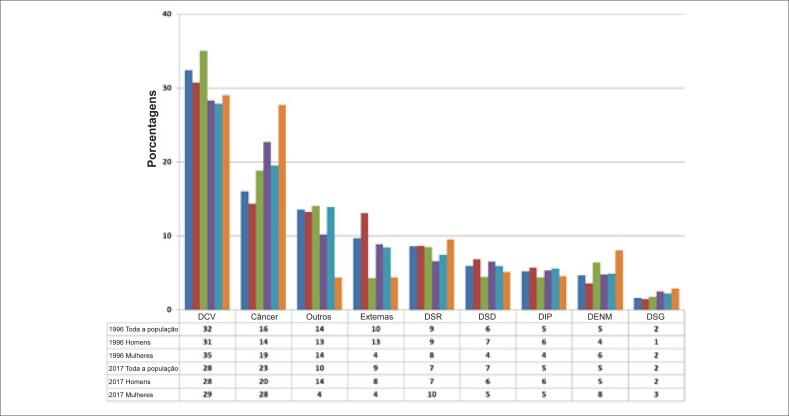
Frequências das seis principais causas de morte na população brasileira. DCV: doenças cardiovasculares; DSD: doenças do sistema digestivo; DENM: doenças endócrinas, nutricionais e metabólicas; Externas: causas externas de morbimortalidade; DSG: doenças do sistema geniturinário; DIP: algumas doenças infecciosas e parasitárias; DSR: doenças do sistema respiratório; Outros: sinais, sintomas, e achados clínicos e laboratoriais anormais sem outra classificação.

A frequência das seis principais causas de morte na população brasileira está apresentada na Figura 2. As porcentagens de mortes por DCV diminuíram, e por neoplasias aumentaram na população geral, tanto em homens como em mulheres, de 1996 a 2017. Nos anos de 1996 e 2017, as DCV e as neoplasias foram causas de, respectivamente, 48,4% e 51,0% das mortes na população geral, 45,0% e 47,4% em homens e 53,8% e 56,7% nas mulheres. A taxa de mortalidade por todas as causas, ajustada por idade (35 a 74 anos), está descrita na Tabela 1. Observamos uma redução de 28% na taxa de mortalidade por todas as causas ajustada por idade na população geral (VPAM = −1.6%; p <0,001) e em ambos os sexos (p<0.001). A análise da taxa de mortalidade a cada cinco anos na faixa etária entre 35 e 74 anos mostrou uma redução significativa na mortalidade por todas as causas em todos os grupos na população geral (p<0,001). Também observamos uma redução significativa na mortalidade por todas as causas na população geral (p<0,01) e em ambos os sexos (Tabela 3).

**Tabela 1:** Taxas de mortalidade, ajustadas por idade, por doenças cardiovasculares (DCV), doenças isquêmicas do coração, doenças cerebrovasculares (DCbV), e câncer, por 100 000 pessoas, na população total, homens e mulheres, no Brasil entre 1996 e 2017

População geral	1996	2017	% mudança	VPAM(%)	IC99%		p
Todas as causas de mortes	1032,24	744,67	–28	–1,6	–1,8	–1,4	<0,000
Doença cardiovascular	342,85	211,94	–38	–2,3	–2,5	–2,1	<0,001
Doenças isquêmicas do coração	111,46	79,19	–29	–1,6	–1,8	–1,5	<0,001
DCbV	85,5	38,6	–55	–3,7	–3,9	–3,5	<0,001
Doenças isquêmicas do coração + DCbV	196,96	117,78	–40	–2,4	–2,6	–2,2	<0,001
Câncer	166,13	166,37	0	–0,1	–0,4	0,1	0,2
**Homens**							
Todas as causas de mortes	1327,36	964,96	–27	–1,6	–1,8	–1,3	<0,000
Doença cardiovascular	421,96	272,35	–35	–2,1	–2,3	–1,9	<0,001
Doenças isquêmicas do coração	150,23	110,52	–26	–1,5	–1,7	–1,3	<0,001
DCbV	102,9	49,13	–52	–3,5	–3,7	–3,3	<0,001
Doenças isquêmicas do coração + DCbV	253,13	159,65	–37	–2,2	–2,4	–2	<0,001
Câncer	194,36	187,29	–3,7	–0,3	–0,5	–0,1	<0,001
**Mulheres**							
Todas as causas de mortes	764,76	548,76	–28	–1,7	–1,9	–1,5	<0,000
Doença cardiovascular	270,84	159,26	–41	–2,5	–2,8	–2,3	<0,001
Doenças isquêmicas do coração	76,25	51,63	–32	–1,9	–2,1	–1,7	<0,001
DCbV	69,81	29,66	–57	–3,9	–4,2	–3,7	<0,001
Doenças isquêmicas do coração + DCbV	146,07	81,29	–44	–2,8	–3	–2,5	<0,001
Câncer	141,23	149,38	5,8	0,1	–0,1	0,3	0,4

%mudança: taxa de mortalidade em 2017 menos a taxa de mortalidade em 1996; VPAM: variação percentual anual média; IC: intervalo de confiança.

**Tabela 2:** Taxas de mortalidade (por 100000 pessoas) por doenças cardiovasculares (DCV) e câncer na população geral no Brasil entre 1996 e 2017

	Doenças cardiovasculares						Câncer					
Faixa etária	1996	2017	% mudança	VPAM (%)	IC99%		1996	2017	% mudança	VPAM (%)	IC99%	
35 – 39	47,62	26,36	–45	–2,5*	–2,8	–2,2	26,64	25,65	–4	–0,3*	–0,5	–0,1
40 – 44	88,85	49,41	–44	–2,8*	–3,0	–2,7	49,73	43,99	–12	–0,9*	–1,0	–0,7
45 – 49	153,2	87,01	–43	–2,8*	–3,1	–2,6	86,77	76,26	–12	–0,9*	–1,2	–0,6
50 – 54	245,94	146,86	–40	–2,7*	–3,0	–2,4	135,69	130,53	–4	–0,5*	–0,8	–0,2
55 – 59	394,94	229,14	–42	–2,5*	–2,9	–2,2	212,25	209,39	–1	–0,1	–0,4	0,2
60 – 64	614,22	383,33	–38	–2,2*	–2,4	–2,0	306,56	316,44	3	0,1	–0,1	0,2
65 – 69	936,57	599,92	–36	–2,1*	–2,3	–1,9	431,34	441,09	2	0,1	–0,1	0,3
70 – 74	1449,9	952,99	–34	–2,0*	–2,2	–1,8	566,21	610,74	8	0,2	–0,0	0,4
**Homens**												
35 – 39	57,21	32,28	–44	–2,4*	–2,8	–2,1	21,47	18,25	–15	–1,0*	–1,2	–0,8
40 – 44	109,8	60,55	–45	–2,8*	–3,1	–2,6	44,29	34,95	–21	–1,5*	–1,7	–1,2
45 – 49	188,97	106,62	–44	–2,8*	–3,1	–2,6	87,5	68,5	–22	–1,5*	–1,9	–1,1
50 – 54	188,97	106,62	–44	–2,8*	–3,1	–2,6	146,38	130,76	–11	–0,8*	–1,1	–0,4
55 – 59	501,07	302,72	–40	–2,4*	–2,7	–2,1	248,35	226,81	–9	–0,3	–0,7	0,0
60 – 64	775,24	501,75	–35	–2,0*	–2,3	–1,8	377,57	374,18	–1	–0,1	–0,3	0,1
65 – 69	1151	777,83	–32	–1,9*	–2,0	–1,7	541,39	541,7	0	–0,1	–0,3	0,1
70 – 74	1715,4	1207,8	–30	–1,7*	–1,9	–1,4	725,44	779,78	7	0,2	–0,0	0,5
**Mulheres**												
35 – 39	38,45	20,5	–47	–2,6*	–3,0	–2,3	31,52	32,98	5	0,2	–0,1	0,5
40 – 44	68,57	38,54	–44	–2,9*	–3,1	–2,7	54,91	52,82	–4	–0,4*	–0,6	–0,3
45 – 49	118,44	68,14	–42	–2,8*	–3,1	–2,6	86,06	83,7	–3	–0,4*	–0,7	–0,0
50 – 54	118,44	68,14	–42	–2,8*	–3,1	–2,6	125,55	130,31	4	–0,2	–0,6	0,1
55 – 59	296,71	161,73	–45	–2,7*	–3,1	–2,4	179,35	193,43	8	0,2	–0,1	0,5
60 – 64	468,94	278,6	–41	–2,5*	–2,7	–2,3	243,39	265,39	9	0,3*	0,1	0,5
65 – 69	748,39	449,47	–40	–2,4*	–2,6	–2,1	336,33	356,04	6	0,3*	0,1	0,5
70 – 74	1218,50	751,48	–38	–2,4*	–2,6	–2,1	430,65	477,05	11	0,2	–0,1	0,4

%mudança: taxa de mortalidade em 2017 menos a taxa de mortalidade em 1996; VPAM: variação percentual anual média; IC: intervalo de confiança;

* <0,001

**Tabela 3:** Taxas de mortalidade (por 100 000 pessoas) por todas as causas na população geral no Brasil entre 1996 e 2017

População geral	1996	2017	% mudança	VPAM (%)	IC99%	
35 – 39	462,36	289,21	–37	–1,8*	–2,0	–1,6
40 – 44	577,73	363,58	–37	–2,0*	–2,2	–1,9
45 – 49	757,12	499,16	–34	–2,0*	–2,2	–1,7
50 – 54	1029,54	730,39	–29	–1,8*	–2,1	–1,6
55 – 59	1487,2	1062,17	–29	–1,7*	–1,9	–1,4
60 – 64	2137,66	1614,4	–24	–1,5*	–1,7	–1,3
65 – 69	3118,78	2374,82	–24	–1,5*	–1,7	–1,3
70 – 74	4550,93	3605,81	–21	–1,4*	–1,7	–1,1
**Homens**						
35 – 39	462,36	289,21	–37	–2,0*	–2,2	–1,8
40 – 44	577,73	363,58	–37	–2,2*	–2,3	–2,0
45 – 49	757,12	499,16	–34	–2,0*	–2,3	–1,7
50 – 54	1029,54	730,39	–29	–1,8*	–2,1	–1,5
55 – 59	1487,2	1062,17	–29	–1,6*	–2,0	–1,3
60 – 64	2137,66	1614,4	–24	–1,4*	–1,7	–1,1
65 – 69	3118,78	2374,82	–24	–1,4*	–1,6	–1,1
70 – 74	4550,93	3605,81	–21	–1,2*	–1,5	–0,9
**Mulheres**						
35 – 39	180,12	125,12	–30	–1,5*	–1,8	–1,2
40 – 44	260,07	178,54	–31	–1,8*	–2,0	–1,6
45 – 49	388,86	267,28	–31	–1,9*	–2,1	–1,6
50 – 54	568,95	400,78	–30	–1,9*	–2,2	–1,6
55 – 59	855,6	599,49	–30	–1,7*	–2,0	–1,4
60 – 64	1273,52	928,53	–27	–1,6*	–1,8	–1,4
65 – 69	1951,63	1408,09	–28	–1,6*	–1,8	–1,4
70 – 74	3031,34	2257,57	–25	–1,6*	–1,8	–1,3

%mudança: taxa de mortalidade em 2017 menos a taxa de mortalidade em 1996; VPAM: variação percentual anual média; IC: intervalo de confiança;

* p<0,001.

### Doenças cardiovasculares

**Figura 3:**
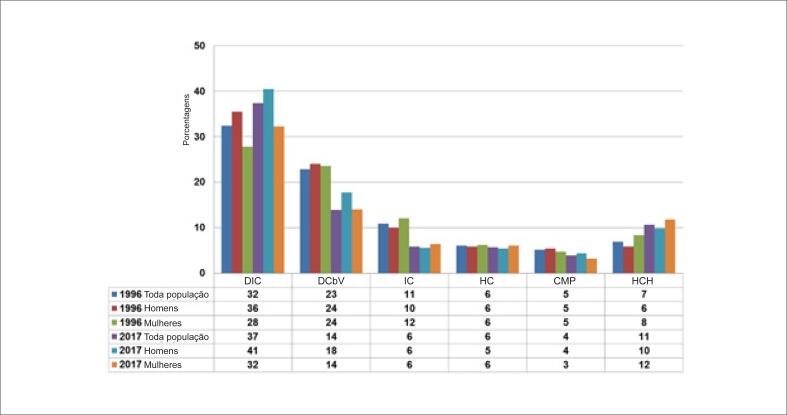
Frequência das seis principais causas de morte por doenças cardiovasculares na população brasileira. DCbV: doenças cerebrovasculares; HC: hemorragia cerebral; CMP: cardiomiopatia; IC: insuficiência cardíaca; HCH: hipertensão e cardiopatia hipertensiva; DIC: doenças isquêmicas do coração.

**Figura 4:**
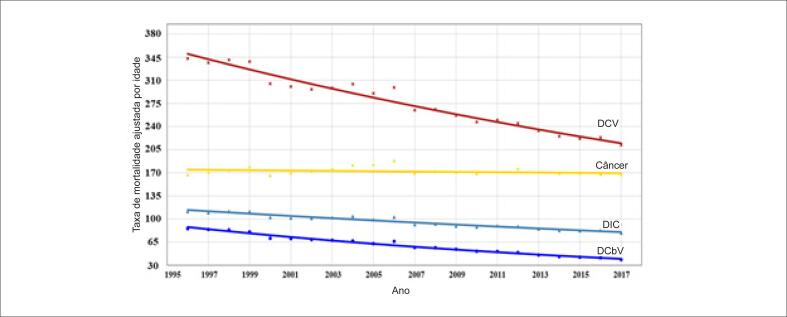
Tendências nas taxas de mortalidade por doenças cardiovasculares (DCV), doenças isquêmicas do coração (DIC), doenças cerebrovasculares (DCbV) e câncer no Brasil entre 1996 e 2017.

**Figura 5:**
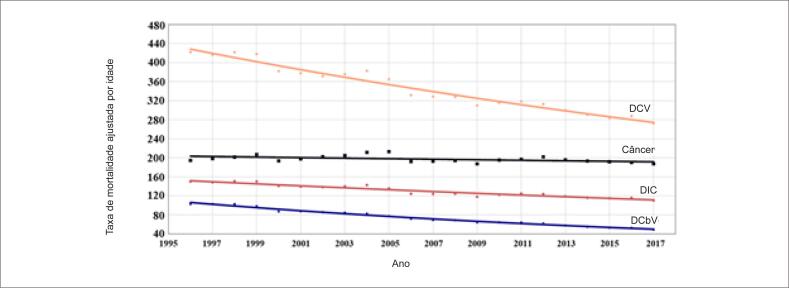
Tendências nas taxas de mortalidade por doenças cardiovasculares (DCV), doenças isquêmicas do coração (DIC), doenças cerebrovasculares (DCbV) e câncer nos indivíduos do sexo masculino no Brasil, entre 1996 e 2017.

As frequências das principais causas de morte por DCV na população geral são apresentadas na Figura 3. Nos anos 1996 e 2017, DIC e DCbV foram responsáveis, respectivamente, por 55,3% e 51,3% de mortes por DCV na população geral, 59,5% e 58,2% em homens e 51,4% e 46,2% nas mulheres. A taxa de mortalidade por DCV, ajustada por idade (35 a 74 anos) está apresentada na Tabela 1. A taxa de mortalidade por DCV ajustada por idade correspondeu, em média, a 31% de mortes por todas as causas, diminuindo de 33% em 1996 a 28% em 2017. As principais causas de morte por DCV foram DIC (média de 35% de mortes por DCV), aumentando de 33% em 1996 para 37% em 2017, seguida de DCbV (média de 22% de mortes por DCB), aumentando de 18% em 1996 para 25% em 2017. A DIC e o DCbV corresponderam a uma média de 57% das DCV no período entre 1996 e 2017 (Tabela 1; Figura 4). Comparações das inclinações da reta de regressão ajustada por idade de DIC versus DCbV mostrou uma maior redução na mortalidade por DCbV (−1,58 vs. −2,25; p<0,001). Observamos uma redução significativa na taxa de mortalidade ajustada por idade para DCV, DIC e DCbV na população geral, em ambos os sexos (p<0,0001 para todos) (Tabela 1; Figuras 5 e 6). A taxa de mortalidade por DIC e DCbV, ajustada por idade, foi respectivamente duas vezes e 1,5 vezes maior em homens em comparação a mulheres. Contudo, comparações de regressões lineares entre homens e mulheres mostraram uma maior redução na taxa de mortalidade por DCV, DIC, e DCbV em homens (p <0,0001).

**Figura 6:**
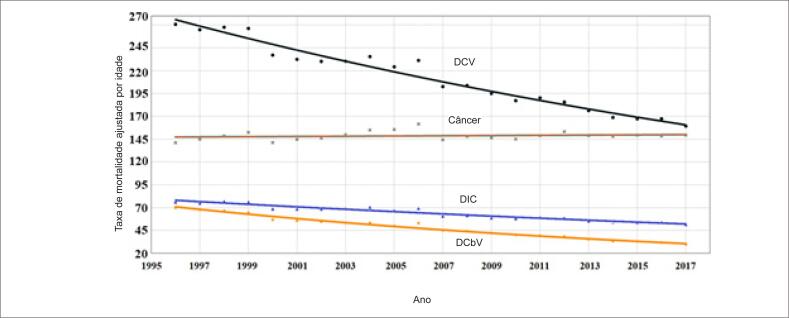
Tendências nas taxas de mortalidade por doenças cardiovasculares (DCV), doenças isquêmicas do coração (DIC), doenças cerebrovasculares (DCbV) e câncer nos indivíduos do sexo feminino no Brasil, entre 1996 e 2017.

A análise da taxa de mortalidade a cada cinco anos no grupo etário entre 35 e 74 anos mostrou uma redução significativa em todos os grupos para mortes por DCV, DIC, e DCbV na população geral (p<0,001) e em ambos os sexos. A redução foi maior para DCbV em comparação à DIC (Tabelas 2 e 4). Observou-se uma redução significativa na taxa de mortalidade por DCV, DIC, e DCbV para todas as idades.

**Tabela 4:** Taxas de mortalidade (por 100 000 pessoas) por doenças isquêmicas do coração e doenças cerebrovasculares na população geral no Brasil entre 1996 e 2017

	Doença isquêmica do coração						Doença cerebrovascular					
	1996	2017	% mudança	VPAM (%)	IC99%		1996	2017	% mudança	VPAM (%)	IC99%	
35 – 39	13.29	8.07	–39	–1,7*	–2,2	–1,2	7,75	2,59	–67	–4,6*	–5,1	–4,1
40 – 44	26.61	17.66	–34	–2,1*	–2,4	–1,8	16,41	6,07	–63	–5,0*	–5,3	–4,7
45 – 49	49.47	32.99	–33	–2,1*	–2,3	–1,9	32,28	10,93	–66	–5,3*	–5,5	–5,0
50 – 54	81.50	58.58	–28	–1,9*	–2,2	–1,6	55,99	19,51	–65	–5,1*	–5,3	–4,8
55 – 59	134.79	93.00	–31	–1,7*	–2	–1,4	94,01	35,01	–63	–4,5*	–4,7	–4,3
60 – 64	211.01	151.31	–28	–1,5*	–1,7	–1,3	149,57	67,34	–55	–3,8*	–4,0	–3,6
65 – 69	307.56	223.24	–27	–1,5*	–1,6	–1,3	247,26	122,81	–50	–3,3*	–3,5	–3,1
70 – 74	443.13	326.20	–26	–1,6*	–1,8	–1,3	415,42	220,28	–47	–2,9*	–3,1	–2,7
**Homens**												
35 – 39	18,95	11,53	–39	–1,8*	–2,3	–1,2	8,16	2,91	–64	–4,5*	–5,1	–4,0
40 – 44	39,03	25,05	–36	–2,3*	–2,7	–2,0	18,52	6,59	–64	–5,1*	–5,5	–4,8
45 – 49	70,45	46,04	–35	–2,2*	–2,5	–2,0	36,38	11,75	–68	–5,4*	–5,7	–5,0
50 – 54	117,12	84,66	–28	–1,8*	–2,1	–1,6	64,97	23,53	–64	–5,0*	–5,3	–4,7
55 – 59	188,45	134,54	–29	–1,6*	–1,9	–1,3	116,25	44,08	–62	–4,5*	–4,7	–4,2
60 – 64	287,28	212,78	–26	–1,3*	–1,6	–1,0	186,52	87,85	–53	–3,7*	–3,9	–3,5
65 – 69	403,3	307,46	–24	–1,2*	–1,4	–1,0	305,16	160,48	–47	–3,1*	–3,3	–2,8
70 – 74	556,69	439,34	–21	–1,1*	–1,3	–0,9	495,87	285,93	–42	–2,6*	–2,8	–2,4
**Mulheres**												
35 – 39	7,92	4,65	–41	–1.8*	–2,5	–1,0	7,35	2,28	–69	–4,7*	–5,2	–4,1
40 – 44	14,76	10,44	–29	–1.8*	–2,1	–1,4	14,4	5,57	–61	–4,8*	–5,2	–4,5
45 – 49	29,43	20,46	–30	–1.9*	–2,2	–1,5	28,36	10,13	–64	–5,1*	–5,5	–4,7
50 – 54	47,72	34,04	–29	–1.9*	–2,3	–1,5	47,48	15,73	–67	–5,2*	–5,6	–4,8
55 – 59	85,91	54,97	–36	–1.8*	–2,5	–1,2	73,75	26,7	–64	–4,6*	–4,9	–4,3
60 – 64	143,16	96,96	–32	–1.9*	–2,1	–1,7	143,16	96,96	–32	–4,0*	–4,2	–3,8
65 – 69	224,89	152,05	–32	–1.8*	–2,1	–1,6	197,26	90,97	–54	–3,6*	–3,9	–3,3
70 – 74	346,44	236,73	–31	–2.1*	–2,3	–1,8	346,93	168,36	–51	–3,2*	–3,5	–2,9

%mudança: taxa de mortalidade em 2017 menos a taxa de mortalidade em 1996; VPAM: variação percentual anual média; IC: intervalo de confiança;

* p<0,001

### Câncer

A taxa de mortalidade por câncer ajustada para idade permaneceu inalterada de 1996 a 2017 e correspondeu, em média, a 20% da mortalidade total, aumentando de 16% em 1996 para 22% em 2017. A mortalidade por câncer excedeu a mortalidade por DIC e DCbV no ano de 2002 (Tabela 1; Figura 4).

Nos homens, houve uma redução significativa na taxa de mortalidade por câncer ajustada por idade no período (p<0,001) e correspondeu, em média, a 17% de mortes por todas as causas, variando de 15% em 1996 a 19% em 2017. A taxa de mortalidade por câncer em homens superou a taxa de mortalidade por DIC e DCbV em 2008 (Tabela 1; Figura 5).

A taxa de mortalidade ajustada por idade nas mulheres permaneceu inalterada entre 1996 e 2017 e correspondeu, em média, a 23% de todas as mortes, aumentando de 18% em 1996 para 27% em 2017. A taxa de mortalidade por câncer, ajustada por idade, superou a taxa de mortalidade por DIC e DCbV em 1997 (Tabela 1; Figura 6). A comparação da diferença das linhas de regressão linear entre homens [y = 203,12 – 0,50 (R2 = 0,21; p = 0,099)] e mulheres [y = 146,82 + 0,16 (R2 = 0,05; p = 0,276)] para todos os tipos de câncer mostrou diferença estatisticamente significativa (p=0,011), indicando uma tendência descendente para homens e ascendente para mulheres.

**Figura 7:**
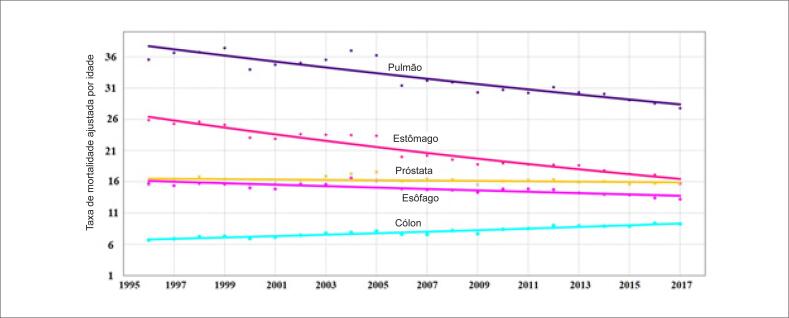
Taxa de mortalidade pelas cinco principais causas de morte por câncer nos indivíduos do sexo masculino no Brasil, entre 1996 e 2017.

**Figura 8:**
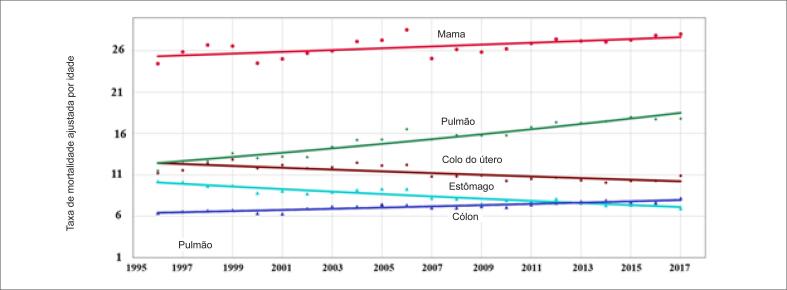
Taxa de mortalidade pelas cinco principais causas de morte por câncer nos indivíduos do sexo feminino no Brasil, entre 1996 e 2017.

As principais causas de morte por câncer nos homens foram câncer de pulmão, estômago, próstata, esôfago e cólon. De 1996 a 2017, observamos uma redução na taxa de mortalidade ajustada por idade por câncer de pulmão, estômago e esôfago e um aumento na taxa de mortalidade ajustada por idade por câncer de cólon. A taxa de mortalidade por câncer de próstata permaneceu inalterada durante o período (Tabela 5; Figura 7). As principais causas de câncer nas mulheres foram câncer de mama, pulmão, cervical, de estômago e de cólon. De 1996 a 2017, houve uma redução na taxa de mortalidade ajustada para idade para câncer de estômago e colo de útero, e um aumento na taxa de mortalidade por câncer de mama, pulmão e cólon (p<0,001) (Tabela 5; Figura 8).

**Tabela 5:** Taxas de mortalidade (por 100000 pessoas) das principais causas de morte por câncer em homens e mulheres no Brasil entre 1996 e 2017

Homens	1996	2017	% mudança	VPAM (%)	IC99%	
Pulmão	35,55	27,78	–22	–1,3*	–1,6	–1,1
Estômago	25,88	15,83	–39	–2,2*	–2,5	–2,0
Próstata	15,51	15,64	1	–0,2	–0,4	0,1
Esôfago	15,73	13,24	–16	–0,8*	–1,0	–0,5
Cólon	6,69	9,26	38	1,5*	1,3	1,8
**Mulheres**						
Mama	24,44	28,01	15	0,4*	0,2	0,6
Pulmão	11,50	17,80	55	1,9*	1,6	2,2
Colo de útero	11,22	10,91	–3	–0,9*	–1,3	–0,6
Estômago	10,22	6,95	–32	–1,6*	–1,9	–1,4
Cólon	6,40	8,20	28	1,0*	0,8	1,3

%mudança: taxa de mortalidade em 2017 menos a taxa de mortalidade em 1996; VPAM: variação percentual anual média; IC: intervalo de confiança;

* p<0,001

A análise da taxa de mortalidade por todos os cânceres em períodos de cinco anos mostrou uma redução na mortalidade no grupo etário entre 35 e 54 anos na população total e nos homens, e nenhuma alteração entre 55 e 74 anos de idade. Nas mulheres, a taxa de mortalidade por câncer diminuiu nos grupos entre 40 e 49 anos e entre 60 e 69 anos. Para todos os demais grupos, a mortalidade manteve-se inalterada (Tabela 2).

## Discussão

O presente estudo mostrou uma redução gradual e persistente na mortalidade por DCV, DIC, e DCbV em homens e mulheres. A redução foi mais acentuada nos homens que nas mulheres.

### Doenças cardiovasculares

O declínio na mortalidade por DCV no Brasil foi similar à observada em países desenvolvidos e em muitos países em desenvolvimento. A redução na mortalidade foi mais significativa em países com maior índice sociodemográfico.^13^ Apesar da redução significativa na mortalidade por DCV no período entre 1996 e 2017, a taxa de mortalidade por DCV nos grupos etários entre 35 e 74 anos no Brasil permaneceu maior que em outros países. Em 2017, a taxa de mortalidade em homens brasileiros foi próxima à observada em homens nos EUA na última atualização da *American Heart Association* (AHA)^14^ Os países com as maiores taxas de mortalidade por DCV em homens foram Bielorrússia, Ucrânia, Rússia, Romênia, Hungria, Sérvia, Eslováquia, Croácia, e República Checa. A taxa de mortalidade nas mulheres por DCV no Brasil em 2017 foi ainda pior se comparada à taxa de mortalidade nos homens, ficando atrás somente da Ucrânia, da Rússia, da Sérvia, e da Romênia segundo última atualização de estatísticas da AHA.^4^ Estudo prévio na população brasileira mostrou uma estabilização na tendência da mortalidade por DIC de 2007 a 2012.^4^ Essa mesma tendência de estabilização nas taxas de morte por DIC foi observada em outros países e foi associada com uma incidência maior de obesidade e diabetes na população.^15^^,^^16^ Estima-se que um em cada dois indivíduos será obeso até o ano de 2030 nos EUA.^17^ Acredita-se que o aumento na incidência desses fatores de risco foi responsável pela desaceleração na tendência descendente de mortes por DCV nos EUA no período entre 2010 e 2017.^18^ Nossos dados, no entanto, indicaram que no Brasil, a partir de 2013, ocorreu uma retomada da tendência descendente na taxa de mortes por DCV, provavelmente devido a uma redução na prevalência de fumantes e melhor controle da hipertensão.

### Câncer

A tendência nas taxas de mortalidade por todos os cânceres permaneceu inalterada entre 1996 e 2017. As principais causas de morte em mulheres foram câncer de mama, pulmão, colo do útero, e estômago de 1996 a 2012, e câncer de cólon de 2013 a 2017. Houve uma tendência crescente nas taxas de mortalidade por câncer de mama, pulmão e cólon, e uma tendência decrescente nas taxas de mortalidade por câncer de estômago. As principais causas de morte por câncer em homens foram câncer de pulmão, estômago, próstata e esôfago, com tendências decrescentes nas taxas de mortalidade por câncer de estômago e pulmão, mas tendência inalterada na mortalidade por câncer de próstata e esôfago. As principais causas de morte por câncer, mas não as tendências nas taxas de mortalidade, foram próximas às observadas em países desenvolvidos, em que câncer de pulmão foi a principal causa de morte, seguida de câncer de próstata em homens e câncer de mama em mulheres.^19^^–^^22^ Desde 1990 e ao contrário do que foi observado no Brasil, houve uma redução na taxa de mortalidade pelos principais tipos de câncer em homens (câncer de pulmão, próstata e cólon) e mulheres (câncer de pulmão, mama, e cólon) nos EUA. A análise mais recente da mortalidade por câncer nos EUA mostrou uma redução significativa de 2,2% entre 2016 e 2017, atribuída, em grande parte, à redução na mortalidade por câncer de pulmão.^23^ Tais variações nas taxas de mortalidade devem-se provavelmente a diferentes tipos e níveis de exposição a carcinógenos, e disponibilidade de serviços de imagens para um diagnóstico precoce. A mesma tendência descendente na mortalidade por todos os tipos de câncer foi observada em homens de 53 de 60 países e em mulheres de 54 de 60 países países de acordo com dados da OMS de 2000 a 2010.^24^ Por outro lado, este estudo mostrou que o Brasil foi um dos poucos países onde a mortalidade por todos os tipos de câncer não diminuiu, e segundo os nossos dados, essa tendência persistiu até (pelo menos) o ano de 2017.

### Doenças cardiovasculares e câncer

Este estudo mostrou que as mortes por DCV e câncer corresponderam a aproximadamente 50% de todas as mortes no período entre 1996 e 2017. Houve uma tendência decrescente na mortalidade por DCV, enquanto as taxas de mortalidade por todos os tipos de câncer permaneceram inalteradas. Um estudo prévio mostrou a mesma tendência de redução da mortalidade por DCV e manutenção da tendência na mortalidade por câncer no Brasil. Em países mais desenvolvidos, no entanto, além da redução da mortalidade por DCV, observou-se também uma redução na mortalidade por câncer.^25^ Uma convergência da mortalidade por essas doenças também foi observada no mundo. Nossos dados mostraram que a mortalidade por DCV no Brasil em 1996 foi duas vezes a mortalidade por câncer, enquanto em 2017, a mortalidade por DCV foi somente 22% maior que a mortalidade por câncer. No entanto, em alguns países desenvolvidos, a mortalidade por câncer já era maior que a mortalidade por DCV. Um estudo recente mostrou que a mortalidade por câncer entre 1999 e 2017 foi maior que por doença cardíaca nos EUA na faixa de idade entre 45 e 64 anos.^7^ A mesma tendência foi observada em muitos países europeus.^26^ Nosso estudo também mostrou que, desde 2002, a mortalidade por câncer foi maior que a soma de mortes por DIC e DCbV. Essa tendência ocorreu mais cedo nas mulheres, em 1997, e mais tarde nos homens, em 2008. Apesar de câncer ter sido a principal causa de mortes em vários países nesse período, observou-se uma tendência de diminuição da mortalidade por todos os tipos de câncer na maioria desses países. Tal fato não foi observado no Brasil, onde as taxas de mortalidade por câncer permaneceram inalteradas.

### Limitações do estudo

A baixa qualidade dos dados de mortalidade no Brasil, exemplificada por erros relacionados ao diagnóstico e à acurácia dos certificados de óbito, a mortes associadas a causas desconhecidas, além de erros na entrada de dados foram as principais limitações do estudo. O número de certificados de óbito com diagnósticos baseados em sintomas, sinais, e achados clínicos e laboratoriais anormais, e não na CID, é um indicador indireto das limitações da qualidade dos dados. Apesar da melhora progressiva, tais certificados são ainda significativos nas regiões nordeste, norte e central, e bem menos nas regiões sul e sudeste do Brasil. Ainda, estudos de validação de dados de mortalidade não são disponíveis na maioria dos estados e cidades do país.

## Conclusão

A população brasileira apresenta diferentes tendências nas taxas de mortalidade por DCV e câncer. As DCV ainda são as principais causas de morte no país, mas, se as tendências observadas nas taxas de mortalidade continuarem, em poucos anos, câncer será a principal causa de morte na população brasileira com idade entre 35 e 74 anos. Assim, prevenção primária das DCV e de câncer deveria ser prioridade, intensificando-se o controle dos principais fatores de risco para DCV, o que também afetaria a incidência de novos tipos de câncer, e melhorando o diagnóstico precoce de câncer.
