# Supplementary material for: Taxas de Mortalidade por Doenças Cardiovasculares e Câncer na População Brasileira com Idade entre 35 e 74 Anos, 1996-2017
Source: Arq Bras Cardiol. 2021 Aug 9;117(2):329–40. [Article in Portuguese] doi: 10.36660/abc.20200233 (PMC8395784; doi:10.36660/abc.20200233)
Supplement: Translation

# Translation: Cardiovascular and Cancer Death Rates in the Brazilian Population Aged 35 to 74 Years, 1996-2017

**Authors:** Antonio de Padua Mansur, Desiderio Favarato

**Affiliations:** 1 São PauloSP Brazil Instituto do Coração do Hospital das Clínicas da Faculdade de Medicina da Universidade de São Paulo, São Paulo, SP – Brazil

**Keywords:** Cardiovascular Diseases/mortality, Epidemiology, Mortality, Brazil, Stroke, Neoplasms, Myocardial Ischemia

## Abstract

**Background:**

Cardiovascular diseases (CVD) and cancer are the main causes of death worldwide. These diseases share many risk factors. Control of traditional risk factors for CVD was associated with lower incidence of cancers.

**Objective:**

To analyze CVD and cancer mortality rate trends in Brazilian population aged 35-74 years from 1996 to 2017.

**Methods:**

Crude and age-adjusted death rate trends were analyzed for all causes of death, CVD, and cancer. Data were obtained from mortality database of the Ministry of Health. Joinpoint Regression Program performed analysis of trends and adjustments in death rates. The degree of changes was determined by the average annual percent change (AAPC). Level of statistical significance was set at p <0.05.

**Results:**

Mortality from all causes of death (AAPC=-1.6%; p<0.001), CVD (AAPC=-2.3; p<0.001), ischemic heart disease (IHD) (AAPC=-1.6; p<0.001) and stroke (AAPC=-3.7; p<0.001) declined. Same trends were observed for CVD (p<0.001) in men and women. Death rates from all causes of cancer (AAPC=-0.1; p=0.201), in men (AAPC=-0.1; p=0.193) and in women (AAPC=-0.1; p=0,871) remained unchanged. In 2002, mortality from cancer exceeded the sum of deaths from IHD and stroke. If trends continue, cancer mortality will also exceed mortality from CVD by 2024. In women, death rates from breast, lung and colon cancer increased, and from cervical and gastric cancers decreased. In men, mortality from lung, stomach and esophagus cancer decreased, and from prostate cancer remained unchanged.

**Conclusion:**

CVD are currently the leading cause of death in Brazil, but death rates from cancer will exceed those from CVD in a few years.

## Introduction

Cardiovascular diseases (CVD) and neoplasms are the leading causes of death in Brazil and worldwide.^1^^,^^2^ In 2017, chronic non-communicable diseases (NCD) were responsible for 73.4% of world mortality.^2^ It is believed that more than 85% of premature deaths due to NCD, of people aged 30 to 69 years, occurred in low-income countries.^3^ Ischemic heart disease (IHD) and stroke accounted for approximately 60% of deaths from CVD. A previous study in Brazil showed a downward trend in mortality from CVD from 1980 to 2012.^4^ During this period, stroke mortality reduced more significantly than IHD mortality. Death rate from CVD had important regional variations in Brazil, with the highest rates in the southeast and south regions,^5^ and convergence of mortality from IHD and stroke in the five regions. The convergence of death rates in these regions occurred earlier for stroke, around 1999, and later for IHD in 2007.

According to the World Health Organization (WHO), cancers were the second leading cause of death from NCD in the world population.^3^ In many developed countries, cancers were the leading cause of death in adults under 70 years of age. In the USA, cancer death rate was higher than CVD death rate in the 45 to 64 age group from 1999 to 2017,^6^ and decreased by 27% from 1991 to 2016. From 2007 to 2016, the annual reduction was 1.4% in women and 1.8% in men.^7^ In Brazil, cancers were the second leading cause of death in 2017.^1^

CVD and cancers share some risk factors. The main risk factors for CVD are also associated with higher incidence of cancers. Recent meta-analysis showed that each risk factor for CVD – smoking, hypertension, diabetes, obesity, excessive alcohol consumption, physical inactivity and low socioeconomic status – was associated with higher incidence of cancers.^8^ On the other hand, control of the main risk factors for CVD was associated with a significant reduction in the incidence of cancer.^9^ Therefore, control of CVD risk factors has also a significant impact in reducing cancer death rate.

This study analyzed trends in death rates from all causes, CVD, IHD, stroke and cancer in women and men of the Brazilian population from 1996 to 2017.

## Methods

We analyzed death rate trends from all causes of diseases, CVD, IHD, stroke and cancers in Brazilian men and women from 1996 to 2017. Crude death rate per 100 000 population was analyzed for each five-year age group between 35 to 74 years. We calculated age-adjusted death rate for this age group per 100 000 population for the study period (1996-2017) using the direct method based on WHO world standard population (2000). Mortality data were obtained from the Vital Health Statistics of the Ministry of Health DATASUS, available online at www2.datasus.gov.br.^10^ The causes of death were classified by the 10th revision of the International Classification of Diseases (ICD). CVD were grouped in codes I00 to I99, IHD in codes I20 to I25, stroke in codes I60 to I69 and cancers in codes C00 to C97. The following codes were used for lung, gastric, prostate, esophageal, colon, breast, and cervical cancers, respectively: C34, C15, C61, C15, C18, C50 and C53. The five main causes of death from cancer were analyzed by sex in period from 1996 to 2017.

### Statistical Analysis

We used the statistical program *Joinpoint Regression Program* version 4.7.0.0. from the National Cancer Institute, Division of Cancer Control and Population Sciences for analysis of age-adjusted death rate trends.^11^ The joinpoint analysis was used to identify the year (independent variable) when significant changes in the mortality rate (dependent variable) occurred during the study period. The degree of the changes was determined by the average annual percent change in death rate (AAPC). Slopes of regression lines of CVD versus cancer and IHD versus stroke were compared using the Microsoft Excel 2010 with t-statistic and two tailed t-distribution.^12^ The statistical significance was set at p <0.05. The study did not require ethical approval as mortality data were obtained from a public website and identity of individuals was not known.

## Results

CVD and cancer age-adjusted death rate per 100 000 population corresponded to 50% of the death rate from all causes. CVD and cancer accounted for approximately 30% and 20% of total mortality, respectively (Table 1). CVD mortality decreased by 38% from 1996 to 2017 and cancer mortality remained unchanged (p<0.001 for comparisons of the slopes of regression lines of CVD versus cancer age-adjusted death rate). In 1996, cancer mortality was 52% lower than CVD mortality but 22% lower in 2017. If these trends continue, cancer mortality will equal CVD mortality in the beginning of 2024 (Figure 1) Likewise, the crude death rate analyzed, every 5 years, for the 35 and 74 age group showed that the mortality from CVD was always higher than cancer death rate (Table 2).

**Table 1:** Age-adjusted mortality rates (per 100 000 population) from cardiovascular diseases, ischemic heart disease, stroke, and cancer in total population, men and women, in Brazil from 1996 to 2017

General population	1996	2017	% change	AAPC(%)	99%CI		p
All causes of deaths	1032.24	744.67	–28	–1.6	–1.8	–1.4	<0.000
Cardiovascular disease	342.85	211.94	–38	–2.3	–2.5	–2.1	<0.001
Ischemic Heart disease	111.46	79.19	–29	–1.6	–1.8	–1.5	<0.001
Stroke	85.5	38.6	–55	–3.7	–3.9	–3.5	<0.001
Ischemic heart disease + stroke	196.96	117.78	–40	–2.4	–2.6	–2.2	<0.001
Cancer	166.13	166.37	0	–0.1	–0.4	0.1	0.2
**Men**							
All causes of death	1327.36	964.96	–27	–1.6	–1.8	–1.3	<0.000
Cardiovascular disease	421.96	272.35	–35	–2.1	–2.3	–1.9	<0.001
Ischemic Heart disease	150.23	110.52	–26	–1.5	–1.7	–1.3	<0.001
Stroke	102.9	49.13	–52	–3.5	–3.7	–3.3	<0.001
Ischemic heart disease + stroke	253.13	159.65	–37	–2.2	–2.4	–2	<0.001
Cancer	194.36	187.29	–3.7	–0.3	–0.5	–0.1	<0.001
**Women**							
All causes of death	764.76	548.76	–28	–1.7	–1.9	–1.5	<0.000
Cardiovascular disease	270.84	159.26	–41	–2.5	–2.8	–2.3	<0.001
Ischemic Heart disease	76.25	51.63	–32	–1.9	–2.1	–1.7	<0.001
Stroke	69.81	29.66	–57	–3.9	–4.2	–3.7	<0.001
Ischemic heart disease + stroke	146.07	81.29	–44	–2.8	–3	–2.5	<0.001
Cancer	141.23	149.38	5.8	0.1	–0.1	0.3	0.4

%change: 2017 death rate minus 1996 death rate; AAPC: average annual percentage change; CI: confidence interval.

**Figure 1:** Trends in mortality rates from cardiovascular disease (DCV) and cancers in Brazilian population aged 35 to 74 years from 1996 to 2017.

**Table 2:** Crude mortality rates (per 100 000 population) from cardiovascular diseases (CVD) and cancer in the general population in Brazil from 1996 to 2017

	Cardiovascular disease						Cancer					
Age group	1996	2017	% change	AAPC(%)	99%CI		1996	2017	% change	AAPC(%)	99%CI	
35 – 39	47,62	26,36	–45	–2.5*	–2.8	–2.2	26,64	25,65	–4	–0.3*	–0.5	–0.1
40 – 44	88,85	49,41	–44	–2.8*	–3.0	–2.7	49,73	43,99	–12	–0.9*	–1.0	–0.7
45 – 49	153,2	87,01	–43	–2.8*	–3.1	–2.6	86,77	76,26	–12	–0.9*	–1.2	–0.6
50 – 54	245,94	146,86	–40	–2.7*	–3.0	–2.4	135,69	130,53	–4	–0.5*	–0.8	–0.2
55 – 59	394,94	229,14	–42	–2.5*	–2.9	–2.2	212,25	209,39	–1	–0.1	–0.4	0.2
60 – 64	614,22	383,33	–38	–2.2*	–2.4	–2.0	306,56	316,44	3	0.1	–0.1	0.2
65 – 69	936,57	599,92	–36	–2.1*	–2.3	–1.9	431,34	441,09	2	0.1	–0.1	0.3
70 – 74	1449,9	952,99	–34	–2.0*	–2.2	–1.8	566,21	610,74	8	0.2	–0.0	0.4
**Men**												
35 – 39	57,21	32,28	–44	–2.4*	–2.8	–2.1	21,47	18,25	–15	–1.0*	–1.2	–0.8
40 – 44	109,8	60,55	–45	–2.8*	–3.1	–2.6	44,29	34,95	–21	–1.5*	–1.7	–1.2
45 – 49	188,97	106,62	–44	–2.8*	–3.1	–2.6	87,5	68,5	–22	–1.5*	–1.9	–1.1
50 – 54	188,97	106,62	–44	–2.8*	–3.1	–2.6	146,38	130,76	–11	–0.8*	–1.1	–0.4
55 – 59	501,07	302,72	–40	–2.4*	–2.7	–2.1	248,35	226,81	–9	–0.3	–0.7	0.0
60 – 64	775,24	501,75	–35	–2.0*	–2.3	–1.8	377,57	374,18	–1	–0.1	–0.3	0.1
65 – 69	1151	777,83	–32	–1.9*	–2.0	–1.7	541,39	541,7	0	–0.1	–0.3	0.1
70 – 74	1715,4	1207,8	–30	–1.7*	–1.9	–1.4	725,44	779,78	7	0.2	–0.0	0.5
**Women**												
35 – 39	38,45	20,5	–47	–2.6*	–3.0	–2.3	31,52	32,98	5	0.2	–0.1	0.5
40 – 44	68,57	38,54	–44	–2.9*	–3.1	–2.7	54,91	52,82	–4	–0.4*	–0.6	–0.3
45 – 49	118,44	68,14	–42	–2.8*	–3.1	–2.6	86,06	83,7	–3	–0.4*	–0.7	–0.0
50 – 54	118,44	68,14	–42	–2.8*	–3.1	–2.6	125,55	130,31	4	–0.2	–0.6	0.1
55 – 59	296,71	161,73	–45	–2.7*	–3.1	–2.4	179,35	193,43	8	0.2	–0.1	0.5
60 – 64	468,94	278,6	–41	–2.5*	–2.7	–2.3	243,39	265,39	9	0.3*	0.1	0.5
65 – 69	748,39	449,47	–40	–2.4*	–2.6	–2.1	336,33	356,04	6	0.3*	0.1	0.5
70 – 74	1218,50	751,48	–38	–2.4*	–2.6	–2.1	430,65	477,05	11	0.2	–0.1	0.4

%change: 2017 death rate minus 1996 death rate; AAPC: average annual percentage change; CI: confidence interval;

* p<0.001

### All-cause mortality

The frequency of the six main causes of death in the Brazilian population is shown in Figure 2. The percentage of CVD decreased, and neoplasms increased in the general population, in both men and women, from 1996 to 2017. In 1996 and 2017, CVD and neoplasms accounted for, respectively, 48.4% and 51.0% of deaths in the general population, 45.0% and 47.4% in men and 53.8% and 56.7% in women. The all-cause mortality rate, adjusted for age (35 to 74 years of age), is described in Table 1. We observed a 28% reduction in age-adjusted all-cause mortality rate in the general population (AAPC = −1.6%; p <0.001) and in both sexes (p<0.001).

**Figure 2:** Frequencies of the six main causes of death in the Brazilian population. CVD: Cardiovascular disease; DSD: Diseases of the digestive system; ENMD: Endocrine, nutritional and metabolic diseases; External: External causes of morbidity and mortality; GUSD: Diseases of the genitourinary system; IPD: Certain infectious and parasitic diseases; RSD: Diseases of the respiratory system; Others: Symptoms, signs and abnormal clinical and laboratory findings, not elsewhere classified.

The analysis of the crude death rate every five years from 35 to 74 years of age showed a significant reduction in all-cause mortality in all age groups in the general population (p <0.001) and in both sexes (Table 3).

**Table 3:** Crude mortality rates per 100 000 population from all causes of death in the general population in Brazil from 1996 to 2017

General population	1996	2017	% change	AAPC(%)	99%CI	
35 – 39	462,36	289,21	–37	–1.8*	–2.0	–1.6
40 – 44	577,73	363,58	–37	–2.0*	–2.2	–1.9
45 – 49	757,12	499,16	–34	–2.0*	–2.2	–1.7
50 – 54	1029,54	730,39	–29	–1.8*	–2.1	–1.6
55 – 59	1487,2	1062,17	–29	–1.7*	–1.9	–1.4
60 – 64	2137,66	1614,4	–24	–1.5*	–1.7	–1.3
65 – 69	3118,78	2374,82	–24	–1.5*	–1.7	–1.3
70 – 74	4550,93	3605,81	–21	–1.4*	–1.7	–1.1
**Men**						
35 – 39	462,36	289,21	–37	–2.0*	–2.2	–1.8
40 – 44	577,73	363,58	–37	–2.2*	–2.3	–2.0
45 – 49	757,12	499,16	–34	–2.0*	–2.3	–1.7
50 – 54	1029,54	730,39	–29	–1.8*	–2.1	–1.5
55 – 59	1487,2	1062,17	–29	–1.6*	–2.0	–1.3
60 – 64	2137,66	1614,4	–24	–1.4*	–1.7	–1.1
65 – 69	3118,78	2374,82	–24	–1.4*	–1.6	–1.1
70 – 74	4550,93	3605,81	–21	–1.2*	–1.5	–0.9
**Women**						
35 – 39	180,12	125,12	–30	–1.5*	–1.8	–1.2
40 – 44	260,07	178,54	–31	–1.8*	–2.0	–1.6
45 – 49	388,86	267,28	–31	–1.9*	–2.1	–1.6
50 – 54	568,95	400,78	–30	–1.9*	–2.2	–1.6
55 – 59	855,6	599,49	–30	–1.7*	–2.0	–1.4
60 – 64	1273,52	928,53	–27	–1.6*	–1.8	–1.4
65 – 69	1951,63	1408,09	–28	–1.6*	–1.8	–1.4
70 – 74	3031,34	2257,57	–25	–1.6*	–1.8	–1.3

%change: 2017 death rate minus 1996 death rate; AAPC: average annual percentage change; CI: confidence interval;

* p<0.001

### Cardiovascular diseases

The frequencies of the main causes of death from CVD in the general population are displayed in Figure 3. From 1996 to 2017, the percentage of deaths from IHD increased and from stroke decreased in both men and women. For the years 1996 and 2017, IHD and stroke accounted for, respectively, 55.3% and 51.3% of deaths from CVD in the general population, 59.5% and 58.2% in men and to 51.4% and 46.2% in women. The mortality rate from CVD, adjusted for age (35 to 74 years of age) is shown in Table 1. The age-adjusted death rate from CVD corresponded, on average, to 31% of all causes of death, decreasing from 33% in 1996 to 28% in 2017. The main causes of death from CVD were IHD (average of 35% of deaths from CVD), increasing from 33% in 1996 to 37% in 2017, followed by stroke (average of 22% of deaths from CVD), increasing from 18% in 1997 to 25% in 2017. IHD and stroke corresponded on average to 57% of all CVD in the period from 1996 to 2017 (Table 1; Figure 4). Comparisons of the age-adjusted regression slopes of IHD versus stroke showed higher reduction in stroke death rate (−1.58 vs. −2,25, respectively; p<0.001). We observed a significant reduction in age-adjusted death rate for CVD, IHD and stroke in the general population, in both women and men (p <0.0001) (Table 1; Figures 5 and 6). The age-adjusted death rate for IHD and stroke were two times and 1.5 times higher in men, respectively, compared to women. However, comparisons of linear regressions between men and women showed a greater reduction in death rate from CVD, IHD and stroke in men (p <0.0001 for all comparisons).

**Figure 3:** The percentage of the six main causes of cardiovascular death in the Brazilian population. CH: cerebral hemorrhage; CMP: cardiomyopathy; HF: heart failure; HHC: hypertension and hypertensive cardiopathy; IHD: ischemic heart disease.

**Figure 4:** Trends in mortality rates from cardiovascular disease (CVD), ischemic heart disease (IHD), stroke, and cancer in Brazil from 1996 to 2017.

**Figure 5:** Trends in mortality rates from cardiovascular disease (CVD), ischemic heart disease (IHD), stroke, and cancer in Brazilian men from 1996 to 2017.

**Figure 6:** Trends in the mortality of cardiovascular disease (CVD), ischemic heart disease (IHD), stroke, and cancer in Brazilian women from 1996 to 2017.

The analysis of the crude death rate every five years from 35 to 74 years of age showed a significant reduction in all age groups for deaths from CVD, IHD and stroke in the general population (p <0.001) and in both sexes. The reduction was greater for stroke compared with IHD (Tables 2 and 4) There was a significant reduction in the crude death rate from CVD, IHD and stroke for all age groups.

**Table 4:** Crude mortality rates per 100 000 population from ischemic heart disease and stroke in the general population in Brazil from 1996 to 2017

	Ischemic heart disease						Stroke					
	1996	2017	% change	AAPC(%)	99%CI		1996	2017	% change	AAPC(%)	99%CI	
35 – 39	13.29	8.07	–39	–1,7*	–2,2	–1,2	7,75	2,59	–67	–4.6*	–5.1	–4.1
40 – 44	26.61	17.66	–34	–2,1*	–2,4	–1,8	16,41	6,07	–63	–5.0*	–5.3	–4.7
45 – 49	49.47	32.99	–33	–2,1*	–2,3	–1,9	32,28	10,93	–66	–5.3*	–5.5	–5.0
50 – 54	81.50	58.58	–28	–1,9*	–2,2	–1,6	55,99	19,51	–65	–5.1*	–5.3	–4.8
55 – 59	134.79	93.00	–31	–1,7*	–2	–1,4	94,01	35,01	–63	–4.5*	–4.7	–4.3
60 – 64	211.01	151.31	–28	–1,5*	–1,7	–1,3	149,57	67,34	–55	–3.8*	–4.0	–3.6
65 – 69	307.56	223.24	–27	–1,5*	–1,6	–1,3	247,26	122,81	–50	–3.3*	–3.5	–3.1
70 – 74	443.13	326.20	–26	–1,6*	–1,8	–1,3	415,42	220,28	–47	–2.9*	–3.1	–2.7
**Men**												
35 – 39	18,95	11,53	–39	–1,8*	–2.3	–1.2	8,16	2,91	–64	–4.5*	–5.1	–4.0
40 – 44	39,03	25,05	–36	–2,3*	–2.7	–2.0	18,52	6,59	–64	–5.1*	–5.5	–4.8
45 – 49	70,45	46,04	–35	–2,2*	–2.5	–2.0	36,38	11,75	–68	–5.4*	–5.7	–5.0
50 – 54	117,12	84,66	–28	–1,8*	–2.1	–1.6	64,97	23,53	–64	–5.0*	–5.3	–4.7
55 – 59	188,45	134,54	–29	–1,6*	–1.9	–1.3	116,25	44,08	–62	–4.5*	–4.7	–4.2
60 – 64	287,28	212,78	–26	–1,3*	–1.6	–1.0	186,52	87,85	–53	–3.7*	–3.9	–3.5
65 – 69	403,3	307,46	–24	–1,2*	–1.4	–1.0	305,16	160,48	–47	–3.1*	–3.3	–2.8
70 – 74	556,69	439,34	–21	–1,1*	–1.3	–0.9	495,87	285,93	–42	–2.6*	–2.8	–2.4
**Women**												
35 – 39	7,92	4,65	–41	–1.8*	–2.5	–1.0	7,35	2,28	–69	–4.7*	–5.2	–4.1
40 – 44	14,76	10,44	–29	–1.8*	–2.1	–1.4	14,4	5,57	–61	–4.8*	–5.2	–4.5
45 – 49	29,43	20,46	–30	–1.9*	–2.2	–1.5	28,36	10,13	–64	–5.1*	–5.5	–4.7
50 – 54	47,72	34,04	–29	–1.9*	–2.3	–1.5	47,48	15,73	–67	–5.2*	–5.6	–4.8
55 – 59	85,91	54,97	–36	–1.8*	–2.5	–1.2	73,75	26,7	–64	–4.6*	–4.9	–4.3
60 – 64	143,16	96,96	–32	–1.9*	–2.1	–1.7	143,16	96,96	–32	–4.0*	–4.2	–3.8
65 – 69	224,89	152,05	–32	–1.8*	–2.1	–1.6	197,26	90,97	–54	–3.6*	–3.9	–3.3
70 – 74	346,44	236,73	–31	–2.1*	–2.3	–1.8	346,93	168,36	–51	–3.2*	–3.5	–2.9

%change: 2017 death rate minus 1996 death rate; AAPC: average annual percentage change; CI: confidence interval;

* p<0.001

### Cancers

The age-adjusted mortality rate from cancer remained unchanged from 1996 to 2017, and corresponded, on average, to 20% of total mortality, increasing from 16% in 1996 to 22% in 2017. Mortality from all cancers exceeded mortality from IHD and stroke in the year 2002 (Table 1; Figure 4).

In men, there was a significant reduction in age-adjusted death rate from all cancers in the period (p<0.001) and corresponded, on average, to 17% of all-cause mortality, ranging from 15% in 1996 to 19% in 2017. Mortality rate from all cancers in men exceeded the death rate from IHD and stroke in 2008 (Table 1; Figure 5).

Age-adjusted death rate from cancer among women remained unchanged from 1996 to 2017 and corresponded, on average, to 23% of all deaths, increasing from 18% in 1996 to 27% in 2017. The age-adjusted mortality rate from cancer exceeded the death rate from IHD and stroke in the year of 1997 (Table 1; Figure 6). The comparison of the difference of the linear regression lines between men [y = 203.12 - 0.50 (R2 = 0.21; p = 0.099)] and women [y = 146.82 + 0.16 (R2 = 0.05; p = 0.276)] for all cancers was statistically significant (p = 0.011) showing declining trend for men and an increasing trend for women.

The main causes of deaths from cancer in men were lung, gastric, prostate, esophagus and colon cancer. From 1996 to 2017, we observed a reduction in age-adjusted death rate for lung, gastric and esophageal cancers, and an increase in colon cancer (p<0.001). Death rate from prostate cancer remained unchanged in the period (Table 5; Figure 7).

**Table 5:** Age-adjusted mortality rates (per 100 000 population) of the main causes of death from cancer in men and women in Brazil from 1996 to 2017

Men	1996	2017	% change	AAPC(%)	99%CI	
Lung	35.55	27.78	–22	–1.3*	–1.6	–1.1
Gastric	25.88	15.83	–39	–2.2*	–2.5	–2.0
Prostate	15.51	15.64	1	–0.2	–0.4	0.1
Esophagus	15.73	13.24	–16	–0.8*	–1.0	–0.5
Colon	6.69	9.26	38	1.5*	1.3	1.8
**Women**						
Breast	24.44	28.01	15	0.4*	0.2	0.6
Lung	11.50	17.80	55	1.9*	1.6	2.2
Cervix	11.22	10.91	–3	–0.9*	–1.3	–0.6
Gastric	10.22	6.95	–32	–1.6*	–1.9	–1.4
Colon	6.40	8.20	28	1.0*	0.8	1.3

%change: 2017 death rate minus 1996 death rate; AAPC: average annual percentage change; CI: confidence interval.

* p<0.001

**Figure 7:** Mortality rates of the five main cause of deaths from cancer in Brazilian men from 1996 to 2017.

The main causes of cancer in women were breast, lung, cervical, gastric and colon cancers. From 1996 to 2017, there was reduction in the age-adjusted death rate for cervical and gastric cancers and an increase in breast, lung, and colon cancers (p<0.001) (Table 5; Figure 8).

**Figure 8:** Mortality rates of the four main cause of deaths from cancer in Brazilian women from 1996 to 2017.

The analysis of death rate from all cancers in five-year periods showed a reduction in mortality for the age group between 35 and 54 years, and no change between 55 and 74 years of age in total population and in men. In women, mortality rate from all cancers reduced only for age groups between 40 and 49 years and between 60 and 69 years. For other age groups, mortality remained unchanged (Table 2).

## Discussion

This study showed persistent and gradual reduction in mortality from CVD, IHD and stroke in men and in women. The reduction was more pronounced in men than in women.

### Cardiovascular diseases

The decline in CVD mortality in Brazil was similar to that observed in developed countries and in many developing countries. The reduction in mortality was more significant in countries with a higher sociodemographic index.^13^ Despite the significant reduction in CVD mortality in the period from 1996 to 2017, the death rate from CVD in age groups between 35 and 74 years in Brazil remained higher when compared to other countries. In Brazil, in 2017, the mortality rate in men was close to that the death rate seen in US men in the latest update of the American Heart Association (AHA).^14^ Countries with the highest CVD death rates in men were, in decreasing order, Belarus, Ukraine, Russia, Romania, Hungary, Serbia, Slovakia, Croatia and Czech Republic. CVD death rate in women in Brazil in 2017 was even worse when compared to death rate in men, ranking behind only the Ukraine, Russia, Belarus, Serbia and Romania according to the latest AHA statistical update.^14^ Previous study in Brazilian population showed stabilization in the trend in mortality from IHD from 2007 to 2012.^4^ This same trend stabilization in IHD death rates was observed in other countries and it was associated with increased incidence of obesity and diabetes in the population.^15^^,^^16^ It is estimated that one of two individuals will be obese by 2030 in USA.^17^ It is believed that the increase in the incidence of these risk factors was responsible for the slowdown in the downward trend in mortality from CVD in the USA in the period from 2010 to 2017.^18^ Our data, however, indicated that in Brazil, starting from 2013, there was a resumption of the downward trend of CVD death rate, probably resulting from a lower prevalence of smoking and better hypertension control.

### Cancer

Trends in mortality rates from all cancers remained unchanged from 1996 to 2017. The main causes of cancer death in women were breast, lung, cervical and stomach from 1996 to 2012 and colon from 2013 to 2017. Increasing trends in mortality rates from breast, lung and colon cancers and decreasing trends in deaths from stomach cancer were observed. The main causes of deaths from cancer in men were lung, stomach, prostate and esophageal cancer, with decreasing trends in lung and stomach cancer death rates, but unchanged trends in mortality from prostate and esophageal cancers. The main causes of death from cancers, but not the trends in death rates, are close to those observed in developed countries, where lung cancer was the most important cause of death followed by prostate cancer in men and breast cancer in women.^19^^–^^22^ Since 1990 and contrary to what was observed in Brazil, decreasing trends in mortality rates from main cancers were observed in men (lung, prostate and colon cancer) and in women (lung, breast and colon cancer) in the United States. The most recent analysis of the death rate from cancer in USA showed a significant reduction of 2.2% between 2016 and 2017, and attributed, in large part, to the reduction in lung cancer mortality.^23^ These variations in death rates are probably due to different types and levels of exposure to carcinogens, and availability of imaging services for early diagnosis. The same downward trends in mortality from all cancers was observed in men from 53 of 60 countries in women from 54 of 60 countries according to WHO data from 2000 to 2010.^24^ On the other hand, this study showed that Brazil was one of the few countries where mortality from all cancers did not decrease and, according to our data, this trend persisted until (at least) 2017.

### Cardiovascular diseases and cancer

This study showed that deaths from CVD and cancers corresponded to around 50% of all deaths in the period from 1996 to 2017. There was a downward trend in mortality from CVD, while mortality rates from all cancers remained unchanged. Previous study showed the same trend of decreasing mortality from CVD and an unchanged trend of mortality from all causes of cancer in Brazil. In more developed countries, however, in addition to the reduction in mortality from CVD, there was also a reduction in cancer mortality.^25^ Likewise, a significant convergence of mortality from these diseases has been observed globally. Our data showed that CVD mortality in Brazil in 1996 was twice as high as cancer mortality, while in 2017, CVD mortality was only 22% higher than cancer mortality. However, in some developed countries, mortality from cancer was already higher than from CVD. A recent study showed that cancer mortality from 1999 to 2017 was higher than from heart disease in USA in the 45 to 64 age group.^7^ The same trend has been observed in several European countries.^26^ Our study also showed that since 2002, cancer mortality has been greater than the sum of death from IHD and stroke. This trend occurred earlier in women, in 1997, and later in men, in 2008. Although cancer was the main cause of death in several countries in this period, a decreasing trend in mortality from all cancers was observed in most of them, which was not observed in Brazil, where mortality rates from all types of cancer remained unchanged.

### Study limitations

The poor quality of mortality data in Brazil, exampled by errors related to the diagnosis and accuracy of death certificates, deaths associated with unknown causes, and errors in data entry were the main limitations of the study. The number of death certificates with diagnosed based on symptoms, signs, and abnormal clinical and laboratory findings, rather than on the ICD, is an indirect indicator of limitations of data quality. Despite progressive improvements, such certificates are still significantly present in the northeast, north, and central west regions of Brazil, but much less in the south and southeast regions. Validation studies for mortality data are also not available in most states or cities in Brazil.

## Conclusion

The Brazilian population has different trends in mortality rates from CVD and cancer. CVD are still the main causes of death in the country, but if the observed death rate trends continue, in a few years cancers will be the main causes of death in the Brazilian population aged 35-74 years. Therefore, primary prevention of CVD and cancers should be prioritized, by intensifying control of the main risk factors for CVD, which will also affect the incidence of new cancers, and improving the early diagnosis of cancer.
